# Patient Care Technician Staffing and Outcomes Among US Patients Receiving In-Center Hemodialysis

**DOI:** 10.1001/jamanetworkopen.2024.1722

**Published:** 2024-03-08

**Authors:** Laura C. Plantinga, Alexis A. Bender, Megan Urbanski, Clarica Douglas-Ajayi, Jennifer Craft Morgan, Karen Woo, Bernard G. Jaar

**Affiliations:** 1 Division of Rheumatology, Department of Medicine, University of California, San Francisco; 2 Division of Nephrology, Department of Medicine, University of California, San Francisco; 3 Division of Geriatrics and Gerontology, Department of Medicine, Emory University, Atlanta, Georgia; 4 Division of Transplantation, Department of Surgery, Emory University, Atlanta, Georgia; 5 National Association of Nephrology Technicians/Technologists, Dayton, Ohio; 6 Gerontology Institute, Georgia State University, Atlanta; 7 Department of Surgery, University of California, Los Angeles; 8 Division of Nephrology, Department of Medicine, Johns Hopkins University, Baltimore, Maryland; 9 Welch Center for Prevention, Epidemiology, and Clinical Research, Johns Hopkins University, Baltimore, Maryland

## Abstract

**Importance:**

Dialysis patient care technicians (PCTs) play a critical role in US in-center hemodialysis (HD) care, but little is known about the association of PCT staffing with patient outcomes at US HD facilities.

**Objective:**

To estimate the associations of in-center HD patient outcomes with facility-level PCT staffing.

**Design, Setting, and Participants:**

This was a retrospective cohort study, with data analysis performed from March 2023 to January 2024. Data on US patients with end-stage kidney disease and their treatment facilities were obtained from the US Renal Data System. Participants included patients (aged 18-100 years) initiating in-center HD between January 1, 2016, and December 31, 2018, who continued receiving in-center HD for 90 days or more and had data on PCT staffing at their initial treating HD facility.

**Exposure:**

Facility-level patient-to-PCT ratios (number of HD patients divided by the number of PCTs reported by the treating facility in the prior year), categorized into quartiles (highest quartile denotes the highest PCT burden).

**Main Outcomes and Measures:**

Patient-level outcomes included 1-year patient mortality, hospitalization, and transplantation. Associations of outcomes with quartile of patient-to-PCT ratio were estimated using incidence rate ratios (IRRs) from mixed-effects Poisson regression, with adjustment for patient demographics and clinical and facility factors.

**Results:**

A total of 236 126 patients (mean [SD] age, 63.1 [14.4] years; 135 952 [57.6%] male; 65 945 [27.9%] Black; 37 777 [16.0%] Hispanic; 153 637 [65.1%] White; 16 544 [7.0%] other race; 146 107 [61.9%] with diabetes) were included. After full adjustment, the highest vs lowest quartile of facility-level patient-to-PCT ratio was associated with a 7% higher rate of patient mortality (IRR, 1.07; 95% CI, 1.02-1.12), a 5% higher rate of hospitalization (IRR, 1.05; 95% CI, 1.02-1.08), an 8% lower rate of waitlisting (IRR, 0.92; 95% CI, 0.85-0.98), and a 20% lower rate of transplant (IRR, 0.80; 95% CI, 0.71-0.91). The highest vs lowest quartile of patient-to-PCT ratio was also associated with an 8% higher rate of sepsis-related hospitalization (IRR, 1.08; 95% CI, 1.03-1.14) and a 15% higher rate of vascular access–related hospitalization (IRR, 1.15; 95% CI, 1.03-1.28).

**Conclusions and Relevance:**

These findings suggest that initiation of treatment in facilities with the highest patient-to-PCT ratios may be associated with worse early mortality, hospitalization, and transplantation outcomes. These results support further investigation of the impact of US PCT staffing on patient safety and quality of US in-center HD care.

## Key Points

**Question:**

Is dialysis patient care technician (PCT) staffing associated with outcomes among patients receiving in-center hemodialysis?

**Findings:**

This cohort study used administrative data for 236 126 US patients initiating hemodialysis and found that the highest vs lowest quartile of facility-level patient-to-PCT ratio was statistically significantly associated with a 7% higher rate of patient mortality, a 5% higher rate of all-cause hospitalization (specifically, an 8% higher rate of sepsis-related hospitalization and a 15% higher rate of vascular access–related hospitalizations), an 8% lower rate of waitlisting, and a 20% lower rate of transplant.

**Meaning:**

These findings suggest that hemodialysis treatment in facilities with the highest patient-to-PCT ratios may be associated with worse patient outcomes, and further study of the association of PCT staffing with patient safety and quality of US in-center hemodialysis care is warranted.

## Teaser

This cohort study examines the association of facility-level patient care technician staffing with outcomes among patients receiving in-center hemodialysis.

## Introduction

For the nearly 500 000 US patients receiving in-center hemodialysis (HD), quality of care remains suboptimal,^1^ despite increasing focus on pay-for-performance^2^ by the Centers for Medicare & Medicaid Services (CMS), which covers the cost of dialysis ($28.5 billion in 2020^1^) for most US residents. One reason for this continuing struggle may be an overburdened dialysis workforce. As part of a CMS-mandated interdisciplinary team (eFigure 1 in Supplement 1),^2^ dialysis patient care technicians (PCTs) play a frontline role in US HD care. Primarily working under the supervision of registered nurses (RNs), PCTs manage technical aspects of in-center HD, including operating and disinfecting complex dialysis machines and filters and setting up prescribed treatment parameters, but they also gain vascular access via needle insertion or catheters and collect and document patient vital signs. The effectiveness of PCTs in delivering high-quality HD care may be undermined by high levels of turnover, which has likely been exacerbated by COVID-19 pandemic–related turnover and staffing shortages.^3^ In 2022, only approximately one-half of US dialysis PCTs intended to continue working as PCTs; of these, only 69% intended to continue working at the same facility.^4^ Frequent turnover could result in disengagement and burnout, inconsistent training, and, ultimately, reduced quality of delivered care and poor outcomes.^5,6,7^

Because of these concerns, a few states have instituted dialysis staffing mandates.^8^ However, there is sparse, inconsistent evidence regarding the association of PCT staffing with in-center HD patient outcomes.^7,9,10,11,12^ Here, we used national administrative data to examine the associations of patient-to-PCT ratios with incident patient mortality, transplantation, and hospitalization within 1 year of in-center HD start.

## Methods

### Study Population and Data Sources

For this retrospective cohort study, we obtained patient^13^ and facility^14^ data from the US Renal Data System.^1^ The study was declared exempt from human participants review; a waiver of informed consent was issued by the institutional review boards of Emory University and University of California, San Francisco, because the data were anonymous, in accordance with 45 CFR §46. This study followed the Strengthening the Reporting of Observational Studies in Epidemiology (STROBE) reporting guidelines for cohort studies.^15^

We identified 1-year outcomes among 333 297 patients aged 18 to 100 years who initiated in-center HD between January 1, 2016, and December 31, 2018, a time frame chosen to limit the effect of the COVID-19 pandemic. We excluded 81 551 patients who did not continue receiving in-center HD for 90 days or longer and 1991 patients who did not have matching facility data. We further excluded patients who initiated treatment in facilities that did not have a corresponding Annual Facility Survey (2699 facilities), did not report a patient-to-PCT ratio (9966 facilities), reported no in-center HD stations (270 facilities) or patients (310 facilities), or had implausible patient-to-PCT ratios (<1, 183 facilities; >96 [considered the maximum for 4 shifts per day, 6 days per week, 4 patients per shift], 207 facilities) (eFigure 2 in Supplement 1).

### Study Variables

#### Patient-to-PCT Ratio

HD patient census at the facility (ie, the number of patients who, at the end of the survey period, were receiving staff-assisted HD or performing outpatient self-HD) and the number of dialysis PCTs were obtained from Annual Facility Survey data.^14^ Dialysis PCT full-time equivalents were estimated using the following formula: (1 × number of full-time dialysis PCTs on December 31 of the survey year) + (0.5 × number of part-time dialysis PCTs on December 31 of the survey year). The patient-to-PCT ratio was then defined as the HD patient census divided by the number of dialysis PCT full-time equivalents. Patient-to-PCT ratios as reported from the most recent prior Annual Facility Survey (2015-2017) were assigned to patients according to their incident facility (linked via the US Renal Data System facility identification) and primarily categorized as quartiles, with the highest quartile representing the highest number of patients per PCT (ie, the highest PCT burden).

#### Outcomes

For all outcomes, patients were followed up for up to 1 year from their first day of HD until death, transplant, dialysis modality switch, loss to follow-up, discontinuation of dialysis, or recovery of kidney function (eFigure 3 in Supplement 1). The date of death in the US Renal Data System patients file was used to assess mortality. The first all-cause hospitalization was defined as the date of the first hospital admission within 1 year of dialysis start. Patients were considered to have a 30-day readmission if they had an admission within 30 days of their first discharge during the study period (ie, the index admission). We also examined hospitalizations due to specific causes that might be affected by PCT care (fluid overload, sepsis, and vascular access complications), using primary diagnosis codes (eTable 1 in Supplement 1). The first cause-specific hospitalization was defined as the date of the first hospital admission attributed to the cause of interest after the incident dialysis date. The dates of first kidney transplant and of first placement on the deceased donor waitlist were used to assess kidney transplant outcomes.

#### Other Variables

Patient-level variables at dialysis start included incident patient age, sex, race and ethnicity (as reported by the facility), receipt of predialysis nephrology care, diabetes status, initial vascular access type, functional impairment (defined as any of the following: inability to ambulate or transfer, needing assistance with activities of daily living, or institutionalization), and insurance. Data on race and ethnicity were included in this study because of well-known racial and ethnic disparities in HD outcomes. Facility-level variables included ownership (for-profit vs not-for-profit), chain (grouped as large dialysis organization vs not), patient-to-staff ratios (for RNs, all nurses [RNs and licensed practical or vocational nurses who are supervised by RNs and have fewer educational and licensing requirements], and social workers, defined similarly to patient-to-PCT ratios), presence of an advanced practice practitioner (ie, nurse practitioner or physician assistant) or licensed practical nurse or licensed vocational nurse, state or territory (grouped as regions), and number of HD treatments provided on an outpatient basis in the prior year. Treatment-to-PCT ratios were estimated as the number of HD treatments provided during the survey period divided by the dialysis PCT full-time equivalents. Area-level indicators of poverty and education were obtained from 2016 to 2020 American Community Survey data^16^ and linked via zip code tabulation area.

### Statistical Analysis

Analyses were performed between March 15, 2023, and January 9, 2024. Patient and facility characteristics were summarized overall and by quartile of patient-to-PCT ratio using analysis of variance and χ^2^ tests. Cumulative incidence curves were generated using Kaplan-Meier methods. Mixed-effects Poisson models with facility as a random effect were used to estimate incidence rate ratios (IRRs) for outcomes by patient-to-PCT ratio. Patient and facility characteristics that were considered a priori to be associated with patient-to-PCT ratios and outcomes were included in sequentially adjusted models, including demographics (age at index admission, sex, and race), clinical characteristics (diabetes, predialysis nephrology care, and vascular access type), and facility characteristics (number of HD stations, profit status, and US region). Complete case analysis was used for 200 863 patients (eTable 2 in Supplement 1). Analyses stratified by the median value of patient-to-nurse ratio were performed to determine whether overall staffing modified the associations. We also performed several sensitivity analyses. First, we examined characteristics by whether individuals were included in vs excluded from the models due to missing data on covariates. Next, we further adjusted for patient-to-nurse ratio and individual-level and area-level socioeconomic status (SES) indicators. We examined patient-to-PCT ratio excluding outliers (<1st or >99th percentile, using cutoffs of <9, 9-12, and >12, with 9-12 representing 3-4 patients per shift for 3 shifts). As a continuous variable, we defined outcomes as only those events occurring at 90 days after dialysis start or later (hospitalizations and waitlisting) using *International Statistical Classification of Diseases and Related Health Problems, Tenth Revision *codes in any position (vs primary diagnosis only; cause-specific hospitalizations only). Using time-to-event analyses, we included Cox proportional hazards models and Fine-Gray subdistribution hazard models^17^ to account for competing risks and estimated associations with patient-to-PCT ratio to assess for misclassification. All analyses were performed with Stata statistical software version 17.0 (StataCorp). A priori 2-sided statistical significance was set at *P* < .05 for the primary analyses. Secondary and sensitivity analyses were post hoc and should be considered exploratory in the setting of multiple comparisons.

## Results

### Patient and Facility Characteristics by Patient-to-PCT Staffing Ratio

Among 236 126 patients (mean [SD] age, 63.1 [14.4] years; 135 952 [57.6%] male; 65 945 [27.9%] Black; 37 777 [16.0%] Hispanic; 153 637 [65.1%] White; 16 544 [7.0%] other race [American Indian or Alaska Native, Asian, Hawaiian or Pacific Islander, and multiracial]; 146 107 [61.9%] with diabetes), the overall median patient-to-PCT ratio was 10.2 (eFigure 4 in Supplement 1). Those treated in facilities with the highest vs lowest quartile of patient-to-PCT ratio (highest vs lowest PCT burden) were more likely to have diabetes (36 421 patients [62.4%] vs 36 312 patients [61.1%]) and to be functionally impaired (10 927 patients [18.7%] vs 9528 patients [16.0%]), but less likely to have received predialysis nephrology care (37 247 patients [74.5%] vs 38 839 patients [77.3%]) or to start HD with a permanent vascular access (11 541 patients [19.8%] vs 14 083 patients [23.7%]) (Table 1). Patients treated at facilities with the highest vs lowest quartile of patient-to-PCT ratio were also more likely to be treated at facilities that were not-for-profit (7509 patients [12.9%] vs 6849 patients [11.5%]), were non–large dialysis organizations (17 213 patients [29.5%] vs 16 742 patients [28.2%]), had fewer HD stations (mean [SD], 20.4 [8.6] vs 21.1 [8.4] stations), and were in the Northeast (14 549 patients [24.9%] vs 5014 patients [8.4%]) or South (28 358 patients [48.6%] vs 23 407 patients [39.4%]) and had higher mean (SD) patient-to-RN (17.5 [8.6] vs 15.0 [6.6]) and patient-to-social worker (87.8 [34.9] vs 77.6 [35.6]) ratios. Those excluded from models because of incomplete data on covariates were generally similar to those who were included (eTable 2 in Supplement 1).

**Table 1.** Characteristics of US Patients Initiating Hemodialysis From January 1, 2016, to December 31, 2018, and Their Initial Facilities, Overall and by Patient-to-PCT Ratio Quartile^a^

Characteristic	Patients, No. (%)^b^				
	Overall (N = 236 126)	Quartile 1 (n = 59 442)	Quartile 2 (n = 59 264)	Quartile 3 (n = 59 025)	Quartile 4 (n = 58 395)
Patient characteristics					
Age, mean (SD), y	63.1 (14.4)	63.1 (14.4)	62.9 (14.4)	63.1 (14.4)	63.4 (14.3)
Sex at birth					
Female	100 172 (42.4)	25 093 (42.2)	25 147 (42.4)	25 004 (42.4)	24 928 (42.7)
Male	135 952 (57.6)	34 348 (57.8)	34 117 (57.6)	34 020 (57.6)	33 467 (57.3)
Race					
Black	65 945 (27.9)	16 214 (27.3)	16 448 (27.8)	16 708 (28.3)	16 575 (28.4)
White	153 637 (65.1)	38 558 (64.9)	38 215 (64.5)	38 131 (64.6)	38 733 (66.3)
Other^c^	16 544 (7.0)	4670 (7.9)	4601 (7.8)	4186 (7.1)	3087 (5.3)
Ethnicity					
Hispanic	37 777 (16.0)	8760 (14.7)	10 164 (17.2)	10 467 (17.7)	8386 (14.4)
Not Hispanic	198 349 (84.0)	50 682 (85.3)	49 100 (82.9)	48 558 (82.3)	50 009 (85.6)
Received predialysis nephrology care					
Yes	151 915 (75.6)	38 839 (77.3)	37 956 (75.6)	37 873 (74.8)	37 247 (74.5)
No	49 157 (24.4)	11 411 (22.7)	12 259 (24.4)	12 766 (25.2)	12 721 (25.5)
Vascular access at first dialysis					
AVF/AVG	50 941 (21.6)	14 083 (23.7)	12 955 (21.9)	12 362 (21.0)	11 541 (19.8)
Catheter (AVF/AVG maturing)	42 909 (18.2)	10 423 (17.6)	10 248 (17.3)	10 868 (18.4)	11 370 (19.5)
Catheter only	142 042 (60.2)	34 838 (58.7)	36 010 (60.8)	35 742 (60.6)	35 422 (60.7)
Diabetes					
Yes	146 107 (61.9)	36 312 (61.1)	36 630 (61.8)	36 744 (62.3)	36 421 (62.4)
No	90 019 (38.1)	23 130 (38.9)	22 634 (38.2)	22 281 (37.8)	21 974 (37.6)
Functional impairment^d^					
Yes	40 862 (17.3)	9528 (16.0)	9949 (16.8)	10 458 (17.7)	10 927 (18.7)
No	195 264 (82.7)	49 914 (84.0)	49 315 (83.2)	48 567 (82.3)	47 468 (81.3)
Initial facility characteristics					
Ownership					
For-profit	211 644 (89.6)	52 574 (88.5)	53 745 (90.7)	54 439 (92.2)	50 886 (87.1)
Not-for-profit	24 463 (10.4)	6849 (11.5)	5519 (9.3)	4586 (7.8)	7509 (12.9)
Large dialysis organization					
Yes	169 770 (71.9)	42 700 (71.8)	42 898 (72.4)	42 990 (72.8)	41 182 (70.5)
No	66 356 (28.1)	16 742 (28.2)	16 366 (27.6)	16 035 (27.2)	17 213 (29.5)
Type					
Hospital based	7641 (3.2)	2119 (3.6)	1383 (2.3)	1281 (2.2)	2858 (4.9)
Freestanding	228 485 (96.8)	57 323 (96.4)	57 881 (97.7)	57 744 (97.8)	55 537 (95.1)
Total No. of stations, mean (SD)	21.5 (8.8)	21.1 (8.4)	22.6 (9.4)	21.9 (8.5)	20.4 (8.6)
Treatment-to-PCT ratio, mean (SD)	1606.0 (916.7)	1066.6 (603.2)	1365.1 (462.0)	1609.6 (433.8)	2395.9 (1281.3)
PCT positions unfilled, mean (SD), %	3.1 (8.0)	1.7 (5.4)	2.2 (5.8)	3.0 (7.1)	5.4 (11.6)
Has active peritoneal dialysis patients					
Yes	3823 (1.6)	886 (1.5)	892 (1.5)	950 (1.6)	1095 (1.9)
No	232 303 (98.4)	58 556 (98.5)	58 372 (98.5)	58 075 (98.4)	57 300 (98.1)
Patient-to-registered nurse ratio, mean (SD)	17.0 (7.6)	15.0 (6.6)	17.4 (7.0)	18.1 (7.6)	17.5 (8.6)
Patient-to-all nurse ratio, mean (SD)^e^	14.9 (6.6)	14.1 (6.2)	15.9 (6.3)	16.0 (6.6)	13.8 (6.9)
Patient-to-social worker ratio, mean (SD)	86.4 (34.8)	77.6 (35.6)	89.4 (33.5)	91.0 (33.6)	87.8 (34.9)
Advanced practice practitioner present					
Yes	11 542 (4.9)	2865 (4.8)	2889 (4.9)	3066 (5.2)	2722 (4.7)
No	224 584 (95.1)	56 577 (95.2)	56 375 (95.1)	55 959 (94.8)	55 673 (95.3)
Licensed vocational nurse and/or licensed practical nurse present					
Yes	100 167 (42.4)	13 021 (27.0)	20 726 (35.0)	26 108 (44.2)	37 312 (63.9)
No	135 959 (57.6)	43 421 (73.1)	38 538 (65.0)	32 917 (55.8)	21 083 (36.1)
Region					
Northeast	39 741 (16.8)	5014 (8.4)	7648 (12.9)	12 530 (21.2)	14 549 (24.9)
South	99 813 (42.3)	23 407 (39.4)	23 477 (39.6)	24 571 (41.6)	28 358 (48.6)
Midwest	43 936 (18.6)	12 555 (21.1)	11 367 (19.2)	9987 (16.9)	10 027 (17.2)
West/US Territories	52 636 (22.3)	18 466 (31.1)	16 772 (28.3)	11 937 (20.2)	5461 (9.4)

Abbreviations: AVF, arteriovenous fistula; AVG, arteriovenous graft; PCT, patient care technician.

^a^ Quartile 1 denotes a patient-to-PCT ratio of 8.60 or less (lowest burden), quartile 2 denotes a ratio of 8.61 to 10.20, quartile 3 denotes a ratio of 10.21 to 12.33, and quartile 4 denotes a ratio of 12.34 or higher (highest burden). Minimum and maximum values for patient-to-PCT ratios were 1 and 96.

^b^ *P* < .001 by χ^2^ or analysis of variance for all characteristics except sex (*P* = .40).

^c^ Includes American Indian or Alaska Native, Asian, Hawaiian or Pacific Islander, and multiracial.

^d^ Functional impairment is defined as any of the following: inability to ambulate or transfer, needing assistance with activities of daily living, or institutionalization.

^e^ All nurses include registered nurses and licensed vocational nurse or licensed practical nurses.

### Associations of Dialysis PCT Staffing Ratio With Patient Outcomes

#### Mortality

**Figure 1.**
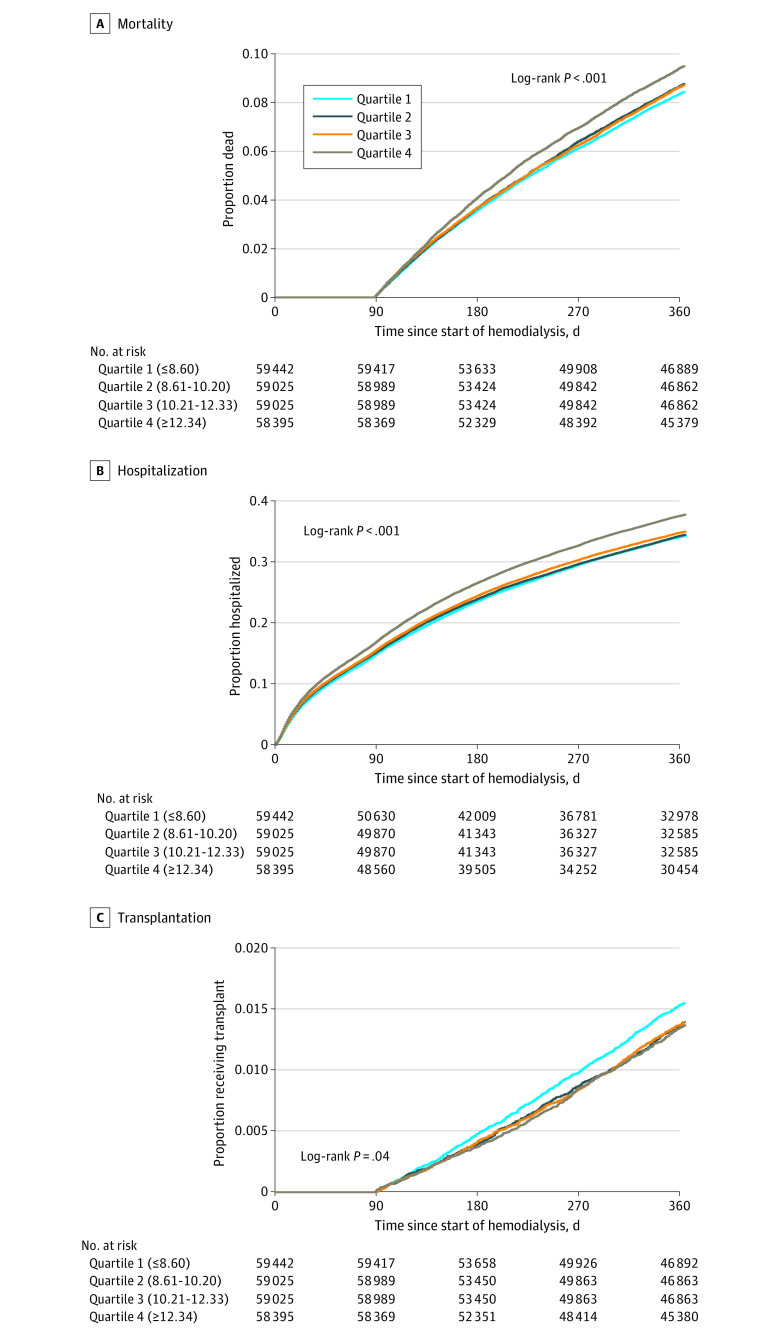
Cumulative Incidence Curves for Mortality, All-Cause Hospitalization, and Transplantation by Quartile of Patient-to-Patient Care Technician Ratio Note that participants who were not receiving in-center hemodialysis for 90 days or more were excluded; thus, mortality and transplantation outcomes that occurred in the first 90 days would not be included in this analysis.

Cumulative incidence of mortality was higher in the fourth (highest) quartile vs the third, second, and first (lowest) quartiles of patient-to-PCT ratio (Figure 1A). In unadjusted models, being treated at facilities with the highest vs lowest quartile was associated with 13% higher 1-year mortality; mortality was 4% higher for patient-to-PCT ratios in the second and third vs first quartile, but the differences were not statistically significant. Adjustment attenuated the associations from an IRR of 1.11 (1.06-1.16) to 1.07 (95% CI, 1.02-1.12) for highest vs lowest quartile, yielding a 7% higher rate of patient mortality after adjustment (Table 2). Results were similar among facilities with patient-to-nurse ratios above and below the median (Table 3). Sensitivity analyses additionally adjusting for patient-to-nurse ratio and SES indicators showed results that were nearly identical to the main analysis (eTable 3 in Supplement 1). Excluding outliers and using prespecified cutoffs showed similar results, although continuous patient-to-PCT ratio was not statistically significantly associated with higher mortality rate (eTable 4 in Supplement 1). Results from time-to-event and time-to-event with competing risks analysis were similar to those from the main analysis (eTable 5 in Supplement 1).

**Table 2.** Associations of Patient-to-PCT Ratio With Patient Outcomes

Outcome and patient-to-PCT ratio quartile^a^	IRR (95% CI)^b^			
	Unadjusted	Adjusted^c^		
		Plus demographics	Plus clinical characteristics	Plus facility characteristics
Mortality				
Quartile 1	1.00 [Reference]	1.00 [Reference]	1.00 [Reference]	1.00 [Reference]
Quartile 2	1.04 (0.99-1.09)	1.04 (1.00-1.10)	1.03 (0.98-1.08)	1.03 (0.98-1.08)
Quartile 3	1.04 (0.99-1.09)	1.04 (0.99-1.09)	1.02 (0.98-1.07)	1.01 (0.96-1.06)
Quartile 4	1.13 (1.08-1.19)^d^	1.12 (1.07-1.18)^d^	1.11 (1.06-1.16)^d^	1.07 (1.02-1.12)^d^
First all-cause hospitalization				
Quartile 1	1.00 [Reference]	1.00 [Reference]	1.00 [Reference]	1.00 [Reference]
Quartile 2	1.02 (0.99-1.05)	1.02 (1.00-1.05)	1.02 (0.99-1.04)	1.01 (0.99-1.04)
Quartile 3	1.03 (1.00-1.06)	1.03 (1.00-1.06)	1.02 (0.99-1.05)	1.00 (0.98-1.03)
Quartile 4	1.12 (1.09-1.15)^d^	1.11 (1.08-1.14)^d^	1.10 (1.07-1.14)^d^	1.05 (1.02-1.08)^d^
First all-cause readmission				
Quartile 1	1.00 [Reference]	1.00 [Reference]	1.00 [Reference]	1.00 [Reference]
Quartile 2	1.03 (0.98-1.08)	1.03 (0.99-1.08)	1.02 (0.98-1.07)	1.01 (0.97-1.06)
Quartile 3	1.04 (0.99-1.09)	1.03 (0.99-1.08)	1.02 (0.98-1.07)	0.99 (0.95-1.04)
Quartile 4	1.14 (1.09-1.20)^d^	1.13 (1.08-1.18)^d^	1.12 (1.07-1.17)^d^	1.05 (1.01-1.10)^d^
First hospitalization due to fluid overload				
Quartile 1	1.00 [Reference]	1.00 [Reference]	1.00 [Reference]	1.00 [Reference]
Quartile 2	1.01 (0.96-1.07)	1.02 (0.97-1.07)	1.01 (0.95-1.06)	1.00 (0.94-1.05)
Quartile 3	1.04 (0.98-1.09)	1.03 (0.98-1.09)	1.02 (0.97-1.08)	0.99 (0.94-1.04)
Quartile 4	1.13 (1.07-1.19)^d^	1.11 (1.05-1.17)^d^	1.10 (1.04-1.16)^d^	1.02 (0.97-1.08)
First hospitalization due to sepsis				
Quartile 1	1.00 [Reference]	1.00 [Reference]	1.00 [Reference]	1.00 [Reference]
Quartile 2	1.02 (0.97-1.07)	1.02 (0.97-1.08)	1.01 (0.96-1.06)	1.01 (0.96-1.06)
Quartile 3	1.05 (1.00-1.11)	1.05 (1.00-1.10)	1.03 (0.98-1.09)	1.02 (0.96-1.07)
Quartile 4	1.16 (1.10-1.22)^d^	1.15 (1.09-1.21)^d^	1.13 (1.08-1.19)^d^	1.08 (1.03-1.14)^d^
First hospitalization due to vascular access complications				
Quartile 1	1.00 [Reference]	1.00 [Reference]	1.00 [Reference]	1.00 [Reference]
Quartile 2	0.97 (0.87-1.09)	0.97 (0.87-1.08)	0.98 (0.88-1.10)	0.98 (0.88-1.09)
Quartile 3	1.07 (0.96-1.20)	1.07 (0.96-1.19)	1.08 (0.97-1.20)	1.05 (0.94-1.17)
Quartile 4	1.21 (1.09-1.35)^d^	1.20 (1.07-1.34)^d^	1.22 (1.09-1.35)^d^	1.15 (1.03-1.28)^d^
First transplant				
Quartile 1	1.00 [Reference]	1.00 [Reference]	1.00 [Reference]	1.00 [Reference]
Quartile 2	0.96 (0.85-1.07)	0.96 (0.86-1.08)	0.97 (0.87-1.09)	0.95 (0.85-1.06)
Quartile 3	0.91 (0.81-1.02)	0.92 (0.82-1.04)	0.95 (0.85-1.06)	0.87 (0.78-0.98)^d^
Quartile 4	0.88 (0.79-0.99)^d^	0.91 (0.80-1.02)	0.93 (0.83-1.05)	0.80 (0.71-0.91)^d^
First transplant waitlisting^e^				
Quartile 1	1.00 [Reference]	1.00 [Reference]	1.00 [Reference]	1.00 [Reference]
Quartile 2	0.99 (0.93-1.06)	1.00 (0.93-1.06)	1.00 (0.94-1.07)	0.99 (0.93-1.06)
Quartile 3	0.95 (0.89-1.01)	0.96 (0.90-1.03)	0.97 (0.91-1.04)	0.94 (0.88-1.00)
Quartile 4	0.92 (0.86-0.99)^d^	0.95 (0.89-1.02)	0.96 (0.90-1.03)	0.92 (0.85-0.98)^d^

Abbreviations: IRR, incidence rate ratio; PCT, patient care technician.

^a^ Quartile 1 denotes a patient-to-PCT ratio of 8.60 or less (lowest burden), quartile 2 denotes a ratio of 8.61 to 10.20, quartile 3 denotes a ratio of 10.21 to 12.33, and quartile 4 denotes a ratio of 12.34 or higher (highest burden). Minimum and maximum values for patient-to-PCT ratios were 1 and 96.

^b^ IRRs are from mixed-effects Poisson models including facility as a random effect (n = 200 863, including individuals with complete data on all covariates).

^c^ Models were adjusted for demographics (age, sex, and race) and clinical characteristics (demographics plus predialysis nephrology care, diabetes, and first vascular access type; plus facility characteristics, demographics plus clinical characteristics plus number of stations, profit status, and region).

^d^ Denotes a statistically significant estimate at *P* < .05.

^e^ Individuals who were waitlisted before hemodialysis start were excluded, leaving 194 842 patients with complete data on all covariates.

**Table 3.** Associations of Patient-to-PCT Ratio With Patient Outcomes, Stratified by Patient-to-All Nurses Ratio

Outcome and patient-to-PCT ratio quartile^a^	IRR (95% CI)^b^	
	Patient-to-nurse ratio ≤14^c^	Patient-to-nurse ratio >14^c^
Mortality		
Quartile 1	1.00 [Reference]	1.00 [Reference]
Quartile 2	1.04 (0.98-1.11)	1.02 (0.95-1.09)
Quartile 3	1.05 (0.98-1.12)	0.99 (0.92-1.06)
Quartile 4	1.06 (0.99-1.13)	1.09 (1.01-1.18)^d^
First all-cause hospitalization		
Quartile 1	1.00 [Reference]	1.00 [Reference]
Quartile 2	1.01 (0.97-1.04)	1.03 (0.99-1.07)
Quartile 3	1.00 (0.97-1.04)	1.01 (0.93-1.06)
Quartile 4	1.06 (1.02-1.10)^d^	1.04 (1.00-1.09)
First all-cause readmission		
Quartile 1	1.00 [Reference]	1.00 [Reference]
Quartile 2	1.01 (0.95-1.08)	1.04 (0.97-1.11)
Quartile 3	1.01 (0.94-1.07)	1.01 (0.94-1.08)
Quartile4	1.07 (1.02-1.17)^d^	1.01 (0.94-1.09)
First hospitalization due to fluid overload		
Quartile 1	1.00 [Reference]	1.00 [Reference]
Quartile 2	1.02 (0.95-1.10)	0.98 (0.91-1.06)
Quartile 3	0.95 (0.88-1.03)	1.02 (0.95-1.10)
Quartile 4	1.01 (0.94-1.08)	1.03 (0.95-1.13)
First hospitalization due to sepsis		
Quartile 1	1.00 [Reference]	1.00 [Reference]
Quartile 2	1.01 (0.94-1.09)	1.02 (0.95-1.10)
Quartile 3	1.02 (0.95-1.09)	1.02 (0.95-1.10)
Quartile 4	1.09 (1.07-1.16)^d^	1.07 (0.99-1.16)
First hospitalization due to vascular access complications		
Quartile 1	1.00 [Reference]	1.00 [Reference]
Quartile 2	1.02 (0.87-1.18)	0.97 (0.87-1.08)
Quartile 3	1.05 (0.90-1.23)	1.07 (0.96-1.19)
Quartile 4	1.04 (0.90-1.20)^e^	1.33 (1.13-1.58)^d^^,^^e^
First transplant		
Quartile 1	1.00 [Reference]	1.00 [Reference]
Quartile 2	1.00 (0.85-1.17)	0.89 (0.76-1.05)
Quartile 3	0.92 (0.78-1.08)	0.83 (0.70-0.98)^d^
Quartile 4	0.84 (0.72-0.98)^d^	0.76 (0.63-0.93)^d^
First transplant waitlisting		
Quartile 1	1.00 [Reference]	1.00 [Reference]
Quartile 2	1.02 (0.93-1.12)	0.94 (0.86-1.03)
Quartile 3	0.98 (0.89-1.08)	0.88 (0.80-0.97)^d^
Quartile 4	0.93 (0.85-1.02)	0.90 (0.81-1.00)^d^

Abbreviations: IRR, incidence rate ratio; PCT, patient care technician.

^a^ Quartile 1 denotes a patient-to-PCT ratio of 8.60 or less (lowest burden), quartile 2 denotes a ratio of 8.61 to 10.20, quartile 3 denotes a ratio of 10.21 to 12.33, and quartile 4 denotes a ratio of 12.34 or higher (highest burden). Minimum and maximum values for patient-to-PCT ratios were 1 and 96.

^b^ IRRs are from mixed-effects Poisson models including facility as a random effect. Analyses were adjusted for age, sex, race, predialysis nephrology care, diabetes, first vascular access type, number of stations, profit status, and region.

^c^ The median value is 14 patients, with greater than 14 vs less than or equal to 14 patients per nurse representing higher vs lower nurse burden.

^d^ Denotes a statistically significant estimate at *P* < .05.

^e^ Denotes a statistically significant interaction at *P* < .05.

#### Hospitalizations

Cumulative incidence of all-cause hospitalization was higher in the fourth quartile vs the third, second, and first quartiles, with 38.3% of patients initiating in facilities in the highest quartile being hospitalized by the end of the first year vs 34.8% of those initiating at facilities in the lowest quartile (Figure 1B). In unadjusted models, being treated at facilities with the highest vs lowest quartile was associated with 12% higher 1-year all-cause hospitalization rates. Adjustment for demographics, clinical characteristics, and facility characteristics attenuated the associations from an IRR of 1.10 (95% CI, 1.07-1.14) to an IRR of 1.05 (95% CI, 1.02-1.08), for a 5% higher rate of hospitalization (Table 2). Results for 30-day readmissions were similar: the highest vs lowest quartile of patient-to-PCT ratio was associated with 14% higher readmission rates in unadjusted models, with attenuation to 5% after adjustment for facility characteristics (Table 2). Results for hospitalizations were similar when stratified by facility patient-to-nurse ratio; patients treated at facilities with lower patient-to-nurse ratios had higher rates of readmission but the interaction was not statistically significant (Table 3). For both all-cause hospitalizations and readmissions, results were similar after additional adjustment (eTable 3 in Supplement 1). Excluding outliers and using prespecified cutoffs showed similar results; continuous patient-to-PCT ratio was associated with higher overall hospitalization rate only (eTable 4 in Supplement 1). Excluding events at 90 or more days resulted in estimates that were not statistically significant (eTable 6 in Supplement 1). Results from time-to-event analyses were similar to the main results (eTable 5 in Supplement 1).

**Figure 2.**
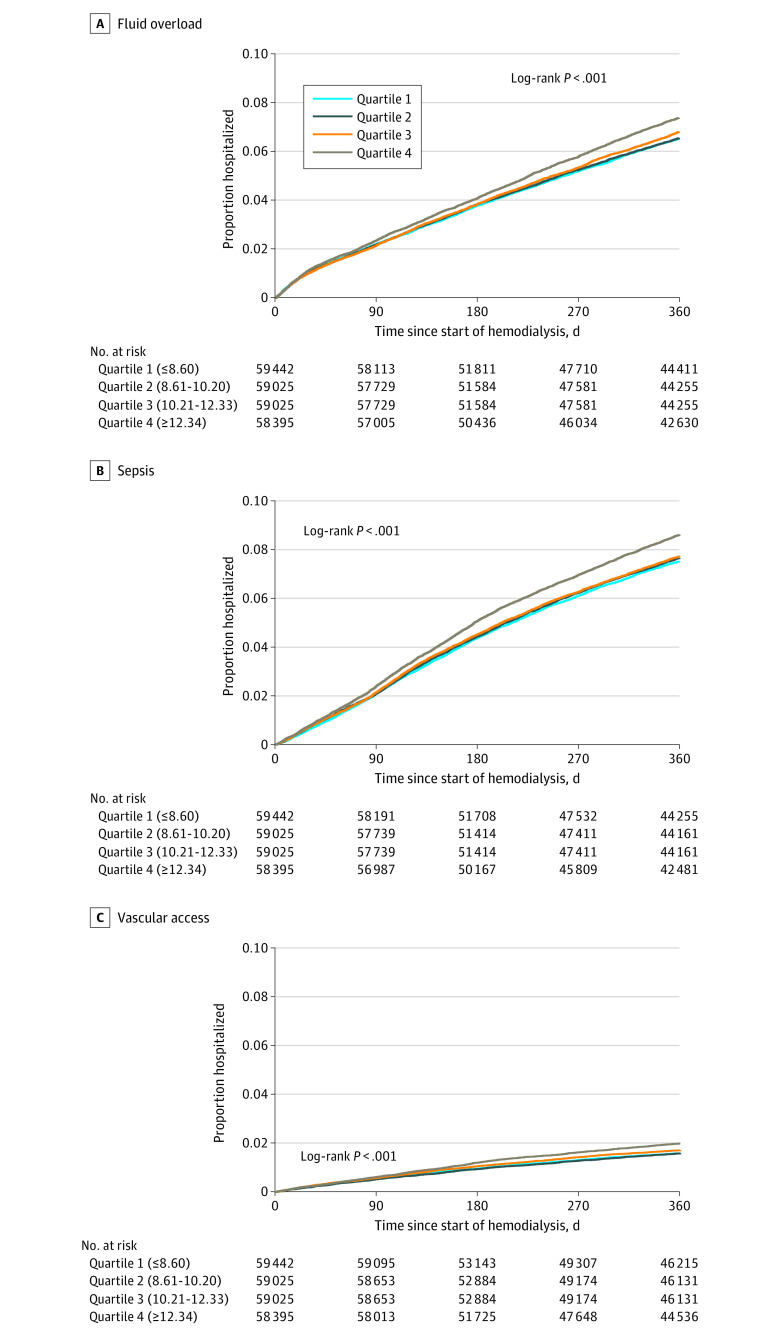
Cumulative Incidence of Cause-Specific Hospitalizations Due to Fluid Overload, Sepsis, and Vascular Access Complications by Quartile of Patient-to-Patient Care Technician Ratio

Results for cause-specific hospitalizations were similar patterns to those for all-cause hospitalizations. For hospitalizations due to fluid overload, sepsis, and vascular access complications, the fourth quartile was associated with higher cumulative incidence than the lower quartiles, regardless of absolute differences in incidence (Figure 2). Unadjusted models using primary diagnostic codes showed that the highest vs lowest quartile was associated with higher rates of hospitalizations due to fluid overload (13% higher), sepsis (16% higher), and vascular access complications (21% higher) (Table 2). After adjustment, only rates of sepsis-related (8% higher rate for highest vs lowest quartile [IRR, 1.08; 95% CI, 1.03-1.14]) and vascular access–related (15% higher rate for highest vs lowest quartile [IRR, 1.15; 95% CI, 1.03-1.28]) hospitalizations remained statistically significantly associated with patient-to-PCT ratio. In stratified analyses, the highest quartile of patient-to-PCT ratio was associated with 33% higher rates of vascular access–related hospitalizations among patients treated at facilities with higher patient-to-nurse ratios, but there was no association in the facilities with lower patient-to-nurse ratios (Table 3). No difference by patient-to-nurse ratio was seen for fluid overload or sepsis-related hospitalizations (Table 3). Sensitivity analyses (eTables 3, 4, 5, and 6 in Supplement 1) were similar to those with overall hospitalizations.

#### Transplantation and Waitlisting

Cumulative incidence of transplantation was lower in the fourth (highest) quartile vs the third, second, and first (lowest) quartiles (Figure 1C). In unadjusted models, being treated at facilities with the highest vs lowest quartile was associated with 12% lower 1-year transplantation rates; transplantation rates were 9% lower for patient-to-PCT ratios in the second quartile and 4% lower for the third vs first quartile, but the differences were not statistically significant. With adjustment, transplantation rates were statistically significantly lower for the third (13% [IRR, 0.87; 95% CI, 0.78-0.98]) and fourth (20% [IRR, 0.80; 95% CI, 0.71-0.91]) vs first quartiles (Table 2). Similarly for transplant waitlisting, the highest vs lowest quartile of patient-to-PCT ratio was statistically significantly associated with 8% lower waitlisting rates (IRR, 0.92; 95% CI, 0.85-0.98) in unadjusted and fully adjusted models, with attenuation after adjustment for demographics and clinical characteristics (Table 2). Results were similar among facilities with patient-to-nurse ratios above and below the median (Table 3). For both transplantation and waitlisting, results with additional adjustment and using time-to-event analysis methods (with and without competing risks) were similar to the main results (eTables 3 and 5 in Supplement 1), as were results excluding events in the first 90 days (eTable 6 in Supplement 1).

## Discussion

In this retrospective cohort study of patients receiving in-center HD, initiating treatment at facilities reporting the highest vs lowest patient burden for PCTs was consistently associated with higher rates of mortality and hospitalization in the first year of treatment and lower rates of waitlisting and transplantation in this population. Many of our observed associations suggested a potential threshold effect, given that associations were primarily seen in the highest vs lowest quartile. To our knowledge, this is one of the first studies to show associations between PCT patient burden and patient outcomes, independent of other patient and facility characteristics.

Our reported associations suggest that reducing patient caseloads for dialysis PCTs could be associated with better outcomes and lower costs. For example, for hospitalization, the difference between 38.3% and 34.8% being hospitalized by the end of the first year receiving in-center HD (the 1-year cumulative rates we observed in the highest and lowest quartiles of patient-to-PCT ratio, respectively) could prevent 7291 hospitalizations in our study population. Given that inpatient expenditures for US patients receiving HD average more than $25 000 per year,^1^ this could result in substantial cost savings. Similarly, higher numbers of transplants could lower costs (annual average per-person expenditures for transplant vs HD in 2019 were approximately $40 000 vs more than $98 000).^1^

The associations we observed do not necessarily reflect the impact of direct PCT care. Patient-to-PCT ratios may be a proxy for other processes of care at dialysis facilities that were not captured in our data, such as poor hiring, training, and retention practices at the facility,^18^ ineffective communication among facility team members,^19,20^ or recent changes in ownership.^9^ However, our results suggest that our associations may be at least partly due to a direct effect. We found that additional adjustment for patient-to-nurse ratio did not change our results, suggesting that the association of the PCT with outcomes was not due to compensatory staffing of RNs, licensed vocational nurses, or licensed practical nurses. In addition, we found that higher patient-to-PCT ratios were more likely to be associated with sepsis-related and vascular access–related hospitalizations—both of which may be affected by the PCT’s attention to infection control protocol, cannulation technique, and observation of patient condition—than with overall hospitalizations. In stratified analyses, we found that the association of high vs low patient-to-PCT ratio with higher vascular access–related hospitalizations was related to facilities with greater patient-to-nurse ratios, which supports the PCT’s role in maintaining the health of the vascular access, particularly in the setting of overburdened nurses. Overall, our results suggest several testable hypotheses for further study.

Importantly, the dialysis PCT is the staff member who spends the most chairside time with the patient in the US in-center HD facility, often leading to deep bonds. These bonds provide an extraordinary opportunity to improve delivery of care and education to patients receiving in-center HD via PCTs, through better, more formal training and supervision. This and future studies can help inform such PCT-targeted interventions. Given worsening dialysis nursing and general workforce shortages,^9,21,22^ the dialysis PCT is needed now more than ever in US HD care. However, there is evidence that PCTs are frequently burned out and considering leaving their facilities or the profession altogether.^4,23^ One of the primary contributing factors cited by PCTs in this burnout survey was the patient caseload.^4^ Thus, addressing PCT staffing issues, potentially through changes such as better pay, more supervisor support, and more respect from other staff,^4^ is of paramount importance.

### Limitations

This study has limitations that should be acknowledged. Misclassification in PCT staffing and/or workload is possible, since we do not know the number of shifts available at facilities or how many shifts PCTs are working. Selection bias is possible in this complete case analysis. There is also the possibility of residual confounding, by unmeasured facility and patient factors, particularly social factors; although adjustment for insurance status and area-level SES indicators did not change our results, these are imperfect measures of individual SES, and these analyses do not rule out the potential for high patient-to-PCT ratios being a marker for more disadvantaged populations. Modest effect sizes and lack of a dose-response for some of our outcomes also weaken causal inference. Misspecification of the model remains a possibility, although our multiple sensitivity analyses showed consistent results, including results in which events occurring before 90 days were excluded to prevent spurious results from early events. There were likely large and multiple shifts in workload starting in 2020 because of the onset of the COVID-19 pandemic, which can be examined in future data; for the purposes of this study, we were interested in the association of PCT staffing with outcomes under typical circumstances.

## Conclusions

Our results suggest that dialysis PCTs may play a critical, often overlooked, role in the quality of care delivered to US patients receiving in-center HD and that higher PCT patient loads may be associated with worse patient outcomes. Further work examining important outcomes in relation to PCT staffing is warranted. In addition, recruitment and retention strategies and strategies to create an environment in which PCTs feel respected and valued may be needed to improve PCT staffing.

## Notes

Supplement 1. **eTable 1.** *ICD-10 *Codes Used in Defining Cause-Specific Hospitalizations **eTable 2.** Characteristics of US Patients Initiating Hemodialysis 1/1/2016-12/31/2018 and Their Initial Facilities, by Inclusion in vs Exclusion From Analytic Models **eTable 3.** Associations of Patient:PCT Ratio With Outcomes Adjusting For Additional Potential Confounders: Sensitivity Analyses **eTable 4.** Associations of Patient:PCT Ratio With Outcomes Using Different Operationalizations of Patient:PCT Ratio: Sensitivity Analyses **eTable 5.** Associations of Patient:PCT Ratio With Outcomes Using Time-to-Event Analyses: Sensitivity Analyses **eTable 6.** Associations of Patient:PCT Ratios With Different Operationalizations of Outcomes: Sensitivity Analyses **eFigure 1.** Mandated US Interdisciplinary Hemodialysis Care Team **eFigure 2.** Selection of Patient Population **eFigure 3. **Patient Follow-Up for Time-to-Event Outcomes **eFigure 4.** Distribution of Patient-Level Patient:PCT Ratio

Supplement 2.
Data Sharing Statement
