# Error in Methods

**DOI:** 10.1001/jamanetworkopen.2024.19682

**Published:** 2024-05-30

In the Original Investigation titled “Patient Care Technician Staffing and Outcomes Among US Patients Receiving In-Center Hemodialysis,”^1^ published March 8, 2024, in the Other Variables subsection of the Methods, the phrase “2016 to 2012 American Community Survey” should have been “2016 to 2020 American Community Survey.” This article has been corrected.^1^

## References

[1] Plantinga LC, Bender AA, Urbanski M, . Patient care technician staffing and outcomes among US patients receiving in-center hemodialysis. JAMA Netw Open. 2024;7(3):e241722. doi:10.1001/jamanetworkopen.2024.1722 38457178 PMC10924248

