# Supplementary material for: Patient Care Technician Staffing and Outcomes Among US Patients Receiving In-Center Hemodialysis
Source: JAMA Netw Open. 2024 Mar 8;7(3):e241722. doi: 10.1001/jamanetworkopen.2024.1722 (PMC10924248; doi:10.1001/jamanetworkopen.2024.1722)
Supplement: Supplement 1. — eTable 1. ICD-10 Codes Used in Defining Cause-Specific Hospitalizations eTable 2. Characteristics of US Patients Initiating Hemodialysis 1/1/2016-12/31/2018 and Their Initial Facilities, by Inclusion in vs Exclusion From Analytic Models eTable 3. Associations of Patient:PCT Ratio With Outcomes Adjusting For Additional Potential Confounders: Sensitivity Analyses eTable 4. Associations of Patient:PCT Ratio With Outcomes Using Different Operationalizations of Patient:PCT Ratio: Sensitivity Analyses eTable 5. Associations of Patient:PCT Ratio With Outcomes Using Time-to-Event Analyses: Sensitivity Analyses eTable 6. Associations of Patient:PCT Ratios With Different Operationalizations of Outcomes: Sensitivity Analyses eFigure 1. Mandated US Interdisciplinary Hemodialysis Care Team eFigure 2. Selection of Patient Population eFigure 3. Patient Follow-Up for Time-to-Event Outcomes eFigure 4. Distribution of Patient-Level Patient:PCT Ratio [file jamanetwopen-e241722-s001.pdf]

Supplemental Online Content

Plantinga LC, Bender AA, Urbanski M, et al. Patient care technician staffing and outcomes among US patients receiving in-center hemodialysis. *JAMA Netw Open*. 2024;7(3):e241722.
doi:10.1001/jamanetworkopen.2024.1722

eTable 1. *ICD-10* Codes Used in Defining Cause-Specific Hospitalizations
eTable 2. Characteristics of US Patients Initiating Hemodialysis 1/1/2016-12/31/2018 and Their Initial Facilities, by Inclusion in vs Exclusion From Analytic Models
eTable 3. Associations of Patient:PCT Ratio With Outcomes Adjusting For Additional Potential Confounders: Sensitivity Analyses
eTable 4. Associations of Patient:PCT Ratio With Outcomes Using Different Operationalizations of Patient:PCT Ratio: Sensitivity Analyses
eTable 5. Associations of Patient:PCT Ratio With Outcomes Using Time-to-Event Analyses: Sensitivity Analyses
eTable 6. Associations of Patient:PCT Ratios With Different Operationalizations of Outcomes: Sensitivity Analyses
eFigure 1. Mandated US Interdisciplinary Hemodialysis Care Team
eFigure 2. Selection of Patient Population
eFigure 3. Patient Follow-Up for Time-to-Event Outcomes
eFigure 4. Distribution of Patient-Level Patient:PCT Ratio

This supplemental material has been provided by the authors to give readers additional information about their work.

© 2024 Plantinga LC et al. *JAMA Network Open.*

eTable 1. *ICD-10* Codes Used in Defining Cause-Specific Hospitalizations

Cause	ICD-10 code(s) included
Fluid overload	E87.70, E87.79 (*fluid overload*)<br>I11.0, I13.2, I50.x (*heart failure*)<br>J81.0, J81.1 (*pulmonary edema*)
Sepsis	A41.9, R65.20, R65.21 (*sepsis*)<br>R78.81 (*bacteremia*)<br>T82.72XXA, T85.79XA (*infection and inflammation due to internal prosthetic devices*)
Vascular access issue	Z49.01 (*catheter placement ([for initial fistula/graft or catheter]*)<br>T82.41xA, T82.42xA, T82.43xA, T82.49xA (*breakdown, displacement, leakage or other complication of initial vascular dialysis catheter*)<br>T82.838A, T82.848A, T82.858A, T82.868A, T82.89A, T92.9xxA (*placement of catheter due to hemorrhage, pain, stenosis, thrombosis or other complications of initial fistula/graft*)<br>T85.81A, T82.510A, T82.520A, T82.530A T82.590A, T82.511A, T82.521A, T82.531A, T82.591A (*breakdown, displacement, leakage, embolism, or other complication of initial fistula/graft*)

© 2024 Plantinga LC et al. *JAMA Network Open.*

eTable 2. Characteristics of US Patients Initiating Hemodialysis 1/1/2016-12/31/2018 and Their Initial Facilities, by Inclusion in vs Exclusion From Analytic Models

**Characteristic**	**Included**	**Excluded**
N	*200,863*	*35,263*
Patient characteristics		
Mean (SD) age	63.2 (14.4)	62.8 (14.3)
Sex at birth, *n* (%)		
Female	85,203 (42.4%)	14,969 (42.4%)
Male	115,660 (57.6%)	20,292 (57.6%)
Race, *n* (%)		
Black	54,542 (27.2%)	11,403 (32.3%)
White	132,232 (65.8%)	21,405 (60.7%)
Other	16,544 (7.0%)	2455 (7.0%)
Ethnicity, *n* (%)		
Hispanic	31314 (15.6%)	6463 (18.3%)
Not Hispanic	169,549 (84.4%)	28,800 (81.7%)
Received pre-dialysis nephrology care, *n* (%)		
Yes	151,752 (75.6%)	163 (78.0%)
No	49,111 (24.5%)	46 (22.0%)
Vascular access at first dialysis, *n* (%)		
AVF/AVG	46,098 (23.0%)	4222 (12.0%)
Catheter (AVF/AVG maturing)	37,865 (18.8%)	5665 (16.2%)
Catheter only	116,900 (58.2%)	25,142 (71.8%)
Diabetes, *n* (%)		
Yes	124,650 (62.1%)	21,457 (60.9%)
No	76,213 (37.9%)	13,806 (39.1%)
Functional impairment, *n* (%)		
Yes	34,207 (17.0%)	6655 (18.9%)
No	166,656 (83.0%)	28,606 (81.1%)
Initial facility characteristics		
Ownership, *n* (%)		
For-profit	178,685 (89.0%)	32,959 (93.5%)
Not-for-profit	22,178 (11.0%)	2285 (6.5%)
Large dialysis organization, *n* (%)		
Yes	142,815 (71.1%)	26,955 (76.4%)
No	58,048 (28.9%)	8308 (23.6%)
Type, *n* (%)		
Hospital-based	7153 (3.6%)	488 (1.4%)
Freestanding	193,710 (96.4%)	34,775 (98.6%)
Total no. of stations, mean (SD)	21.5 (8.8)	21.5 (8.4)
Treatment:PCT ratio, mean (SD)	1613.6 (942.3)	1562.8 (752.7)
% of PCT positions unfilled, mean (SD)	3.1 (8.0)	2.9 (8.0)
Has active PD patients, *n* (%)		
Yes	3076 (1.5%)	747 (2.1%)
No	197,787 (98.5%)	34,516 (97.9%)
Patient:RN ratio, mean (SD)	16.8 (7.5)	17.9 (8.2)
Patient:SW ratio, mean (SD)	86.3 (34.9)	87.2 (34.2)
APP present, *n* (%)		
Yes	9997 (5.0%)	1545 (4.4%)
No	190,866 (95.0%)	33,718 (95.6%)
LVN/LPN present, *n* (%)		
Yes	85,376 (42.5%)	14,791 (41.9%)
No	115,487 (57.5%)	20,472 (58.1%)

© 2024 Plantinga LC et al. *JAMA Network Open.*

Characteristic	Included	Excluded
Region		
Northeast	34,333 (17.1%)	5408 (15.3%)
South	83,932 (41.8%)	15,901 (45.1%)
Midwest	37,936 (18.9%)	6000 (17.0%)
West/U.S. Territories	44,682 (22.3%)	7954 (22.6%)

P<0.001 by chi-square or ANOVA for all except sex (*P*=0.01), pre-dialysis nephrology care (*P*=0.7), and LVN/LPN present (*P*=0.05). APP, advanced practice provider; AVF, arteriovenous fistula; AVG, arteriovenous graft; LVN/LPN, licensed vocational nurse/licensed practical nurse; PCT, patient care technician; Q1-Q4, quartiles 1-4; RN, registered nurse; SW, social worker.

© 2024 Plantinga LC et al. *JAMA Network Open.*

eTable 3. Associations of Patient:PCT Ratio With Outcomes Adjusting For Additional Potential Confounders: Sensitivity Analyses

	Adjusted[a] effect estimate [IRR (95% CI)] by quartile of patient:PCT ratio, additionally adjusting for:			
**Outcome**	**Facility-level patient:all nurses ratio**	**Patient-level insurance[b]**	**Area-level poverty[c]**	**Area-level education[c]**
**Mortality**				
Q1 (≤8.60)	1.00 (ref.)	1.00 (ref.)	1.00 (ref.)	1.00 (ref.)
Q2 (8.61-10.20)	1.03 (0.98-1.08)	1.03 (0.98-1.08)	1.02 (0.97-1.07)	1.02 (0.97-1.07)
Q3 (10.21-12.33)	1.01 (0.97-1.06)	1.03 (0.97-1.06)	1.00 (0.96-1.05)	1.00 (0.96-1.05)
Q4 (>12.34)	1.07 (1.02-1.13)[d]	1.07 (1.02-1.13)[d]	1.06 (1.01-1.12)[d]	1.06 (1.01-1.11)[d]
**First all-cause hospitalization**				
Q1 (≤8.60)	1.00 (ref.)	1.00 (ref.)	1.00 (ref.)	1.00 (ref.)
Q2 (8.61-10.20)	1.01 (0.99-1.04)	1.01 (0.99-1.04)	1.01 (0.99-1.04)	1.01 (0.99-1.04)
Q3 (10.21-12.33)	1.01 (0.98-1.03)	1.00 (0.98-1.03)	1.00 (0.98-1.03)	1.01 (0.98-1.03)
Q4 (>12.34)	1.05 (1.03-1.08)[d]	1.05 (1.02-1.08)[d]	1.05 (1.02-1.08)[d]	1.05 (1.03-1.80)[d]
**First all-cause readmission**				
Q1 (≤8.60)	1.00 (ref.)	1.00 (ref.)	1.00 (ref.)	1.00 (ref.)
Q2 (8.61-10.20)	1.02 (0.97-1.07)	1.02 (0.97-1.06)	1.02 (0.97-1.06)	1.02 (0.97-1.06)
Q3 (10.21-12.33)	1.00 (0.96-1.05)	1.00 (0.95-1.04)	1.00 (0.95-1.04)	0.99 (0.95-1.04)
Q4 (>12.34)	1.06 (1.01-1.11)[d]	1.05 (1.01-1.10)[d]	1.06 (1.01-1.11)[d]	1.06 (1.01-1.11)[d]
**First hospitalization due to fluid overload**				
Q1 (≤8.60)	1.00 (ref.)	1.00 (ref.)	1.00 (ref.)	1.00 (ref.)
Q2 (8.61-10.20)	0.99 (0.94-1.04)	1.00 (0.94-1.05)	1.00 (0.95-1.05)	1.00 (0.95-1.05)
Q3 (10.21-12.33)	0.98 (0.93-1.03)	0.99 (0.94-1.04)	0.99 (0.94-1.05)	0.99 (0.94-1.05)
Q4 (>12.34)	1.02 (0.97-1.08)	1.02 (0.97-1.08)	1.03 (0.97-1.09)	1.03 (0.97-1.09)
**First hospitalization due to sepsis**				
Q1 (≤8.60)	1.00 (ref.)	1.00 (ref.)	1.00 (ref.)	1.00 (ref.)
Q2 (8.61-10.20)	1.01 (0.96-1.06)	1.01 (0.96-1.06)	0.99 (0.94-1.05)	0.99 (0.94-1.05)
Q3 (10.21-12.33)	1.02 (0.97-1.07)	1.02 (0.97-1.07)	1.01 (0.96-1.07)	1.01 (0.96-1.06)
Q4 (>12.34)	1.08 (1.03-1.14)[d]	1.08 (1.03-1.14)[d]	1.07 (1.02-1.13)[d]	1.07 (1.02-1.13)[d]
**First hospitalization due to vascular access complications**				
Q1 (≤8.60)	1.00 (ref.)	1.00 (ref.)	1.00 (ref.)	1.00 (ref.)
Q2 (8.61-10.20)	0.97 (0.87-1.08)	0.98 (0.88-1.09)	0.98 (0.88-1.10)	0.98 (0.87-1.09)
Q3 (10.21-12.33)	1.04 (0.93-1.16)	1.05 (0.94-1.17)	1.06 (0.95-1.19)	1.06 (0.94-1.18)
Q4 (>12.34)	1.14 (1.02-1.28)[d]	1.15 (1.03-1.28)[d]	1.15 (1.03-1.28)[d]	1.14 (1.02-1.28)[d]

© 2024 Plantinga LC et al. *JAMA Network Open.*

Outcome	Adjusted[a] effect estimate [IRR (95% CI)] by quartile of patient:PCT ratio, additionally adjusting for:			
	Facility-level patient:all nurses ratio	Patient-level insurance[b]	Area-level poverty[c]	Area-level education[c]
**First transplant**				
Q1 (≤8.60)	1.00 (ref.)	1.00 (ref.)	1.00 (ref.)	1.00 (ref.)
Q2 (8.61-10.20)	0.95 (0.85-1.06)	0.95 (0.85-1.06)	0.97 (0.87-1.09)	0.97 (0.87-1.08)
Q3 (10.21-12.33)	0.87 (0.78-0.98)[d]	0.88 (0.78-0.98)[d]	0.90 (0.80-1.01)	0.93 (0.80-1.01)
Q4 (>12.34)	0.80 (0.71-0.90)[d]	0.80 (0.71-0.91)[d]	0.83 (0.74-0.94)[d]	0.85 (0.75-0.95)[d]
**First transplant waitlisting**				
Q1 (≤8.60)	1.00 (ref.)	1.00 (ref.)	1.00 (ref.)	1.00 (ref.)
Q2 (8.61-10.20)	0.99 (0.93-1.05)	1.00 (0.93-1.06)	1.00 (0.93-1.06)	1.00 (0.94-1.07)
Q3 (10.21-12.33)	0.93 (0.87-1.00)	0.94 (0.88-1.01)	0.96 (0.90-1.02)	0.96 (0.90-1.03)
Q4 (>12.34)	0.91 (0.85-0.98)[d]	0.92 (0.86-0.98)[d]	0.94 (0.88-1.01)	0.95 (0.88-1.02)

IRR, incidence rate ratio.

[a]Fully adjusted models (including demographics, clinical characteristics, and facility characteristics; see Table 2). From mixed-effects Poisson models including facility as a random effect.

[b]Defined as no vs. any medical coverage at dialysis start.

[c]Area-level poverty and education defined as percentage of households living below the poverty line and percentage of individuals with college degrees, using 2019 American Community Survey data (linked at the zip code level).

[d]Statistically significant estimate at $P<0.05$.

© 2024 Plantinga LC et al. *JAMA Network Open.*

eTable 4. Associations of Patient:PCT Ratio With Outcomes Using Different Operationalizations of Patient:PCT Ratio: Sensitivity Analyses

	Adjusted[a] Effect Estimate (95% CI)				
	Quartiles excluding outliers[b]		Pre-specified cutoffs[c]		Continuous[d]
**Outcome**	**Patient-PCT ratio:**	**IRR (95% CI)**	**Patient-PCT ratio:**	**IRR (95% CI)**	**IRR (95% CI)**
**Mortality**	Q1 (4.80-8.63)	1.00 (ref.)	<9	0.99 (0.95-1.03)	1.004 (1.000-1.007)
	Q2 (8.64-10.20)	1.03 (0.98-1.08)	9-12	1.00 (ref.)	
	Q3 (10.21-12.25)	1.01 (0.97-1.06)	>12	1.05 (1.01-1.09)[e]	
	Q4 (12.26-30.7)	1.07 (1.02-1.13)[e]			
**First all-cause hospitalization**	Q1 (4.80-8.63)	1.00 (ref.)	<9	1.00 (0.98-1.02)	1.002 (1.000-1.004)[e]
	Q2 (8.64-10.20)	1.01 (0.98-1.03)	9-12	1.00 (ref.)	
	Q3 (10.21-12.25)	1.00 (0.97-1.03)	>12	1.04 (1.02-1.06)[e]	
	Q4 (12.26-30.7)	1.05 (1.02-1.07)[e]			
**First all-cause readmission**	Q1 (4.80-8.63)	1.00 (ref.)	<9	1.00 (0.96-1.04)	1.000 (0.997-1.003)
	Q2 (8.64-10.20)	1.02 (0.98-1.08)	9-12	1.00 (ref.)	
	Q3 (10.21-12.25)	1.01 (0.97-1.06)	>12	1.04 (1.00-1.08)	
	Q4 (12.26-30.7)	1.07 (1.02-1.13)[e]			
**First hospitalization due to fluid overload**	Q1 (4.80-8.63)	1.00 (ref.)	<9	1.02 (0.97-1.06)	1.003 (1.000-1.007)
	Q2 (8.64-10.20)	0.99 (0.94-1.05)	9-12	1.00 (ref.)	
	Q3 (10.21-12.25)	0.99 (0.94-1.04)	>12	1.03 (0.98-1.08)	
	Q4 (12.26-30.7)	1.01 (0.95-1.07)			
**First hospitalization due to sepsis**	Q1 (4.80-8.63)	1.00 (ref.)	<9	1.00 (0.95-1.04)	1.003 (1.000-1.007)[e]
	Q2 (8.64-10.20)	1.00 (0.95-1.06)	9-12	1.00 (ref.)	
	Q3 (10.21-12.25)	1.02 (0.97-1.07)	>12	1.06 (1.01-1.10)[e]	
	Q4 (12.26-30.7)	1.08 (1.02-1.13)[e]			
**First hospitalization due to vascular access complications**	Q1 (4.80-8.63)	1.00 (ref.)	<9	0.96 (0.87-1.06)	1.005 (0.998-1.012)
	Q2 (8.64-10.20)	0.97 (0.87-1.08)	9-12	1.00 (ref.)	
	Q3 (10.21-12.25)	1.04 (0.93-1.17)	>12	1.12 (1.02-1.23)[e]	
	Q4 (12.26-30.7)	1.15 (1.03-1.28)[e]			
**First transplant**	Q1 (4.80-8.63)	1.00 (ref.)	<9	1.10 (1.00-1.21)[e]	0.985 (0.977-0.995)[e]
	Q2 (8.64-10.20)	0.95 (0.85-1.07)	9-12	1.00 (ref.)	
	Q3 (10.21-12.25)	0.89 (0.79-1.00)	>12	0.85 (0.77-0.94)[e]	
	Q4 (12.26-30.7)	0.81 (0.72-0.91)			
**First transplant waitlisting**	Q1 (4.80-8.63)	1.00 (ref.)	<9	1.06 (1.00-1.12)[e]	0.994 (0.990-0.999)[e]
	Q2 (8.64-10.20)	0.99 (0.93-1.05)	9-12	1.00 (ref.)	
	Q3 (10.21-12.25)	0.94 (0.88-1.00)	>12	0.96 (0.91-1.02)	
	Q4 (12.26-30.7)	0.92 (0.86-0.99)			

IRR, incidence rate ratio.

© 2024 Plantinga LC et al. *JAMA Network Open.*

[a]Fully adjusted models (including demographics, clinical characteristics, and facility characteristics; see Table 2). From mixed-effects Poisson models including facility as a random effect.
[b]Excluding patient:PCT ratios that are at the 1st percentile (4.8) or lower or 99th percentile (31) or higher.
[c]Determined as 3-4 patients per shift for 3-4 shifts as the reference range.
[d]Per additional patient per PCT.
[e]Statistically significant estimate at *P*<0.05.

© 2024 Plantinga LC et al. *JAMA Network Open.*

eTable 5. Associations of Patient:PCT Ratio With Outcomes Using Time-to-Event Analyses: Sensitivity Analyses

Outcome	Time-to-event analysis[b] HR (95% CI)	Time-to-event with competing risks analysis[b,c] HR (95% CI)
**Mortality**		
Q1 (≤8.60)	1.00 (ref.)	1.00 (ref.)
Q2 (8.61-10.20)	1.03 (0.98-1.08)	1.03 (0.99-1.08)
Q3 (10.21-12.33)	1.02 (0.97-1.06)	1.02 (0.98-1.07)
Q4 (>12.34)	1.07 (1.03-1.12)[d]	1.07 (1.03-1.12)[d]
**First all-cause hospitalization**		
Q1 (≤8.60)	1.00 (ref.)	1.00 (ref.)
Q2 (8.61-10.20)	1.01 (0.98-1.03)	1.01 (0.98-1.03)
Q3 (10.21-12.33)	1.00 (0.98-1.02)	1.00 (0.98-1.03)
Q4 (>12.34)	1.04 (1.02-1.07)[d]	1.04 (1.02-1.07)[d]
**First all-cause readmis ion**		
Q1 (≤8.60)	1.00 (ref.)	1.00 (ref.)
Q2 (8.61-10.20)	1.01 (0.97-1.05)	1.01 (0.97-1.05)
Q3 (10.21-12.33)	0.99 (0.95-1.04)	1.00 (0.96-1.04)
Q4 (>12.34)	1.05 (1.01-1.09)[d]	1.05 (1.01-1.09)[d]
**First hospitalization due to fluid overload**		
Q1 (≤8.60)	1.00 (ref.)	1.00 (ref.)
Q2 (8.61-10.20)	1.00 (0.95-1.05)	1.00 (0.95-1.05)
Q3 (10.21-12.33)	0.99 (0.94-1.04)	0.99 (0.94-1.04)
Q4 (>12.34)	1.02 (0.97-1.08)	1.02 (0.97-1.07)
**First hospitalization due to sepsis**		
Q1 (≤8.60)	1.00 (ref.)	1.00 (ref.)
Q2 (8.61-10.20)	1.01 (0.96-1.05)	1.01 (0.96-1.05)
Q3 (10.21-12.33)	1.01 (0.97-1.06)	1.02 (0.97-1.06)
Q4 (>12.34)	1.09 (1.03-1.14)[d]	1.08 (1.03-1.14)[d]
**First hospitalization due to vascular access complications**		
Q1 (≤8.60)	1.00 (ref.)	1.00 (ref.)
Q2 (8.61-10.20)	0.99 (0.89-1.10)	0.99 (0.89-1.09)
Q3 (10.21-12.33)	1.11 (1.00-1.22)	1.07 (0.97-1.19)
Q4 (>12.34)	1.25 (1.13-1.38)[d]	1.17 (1.05-1.29)[d]
**First transplant**		
Q1 (≤8.60)	1.00 (ref.)	1.00 (ref.)
Q2 (8.61-10.20)	0.93 (0.83-1.03)	0.93 (0.83-1.03)
Q3 (10.21-12.33)	0.87 (0.78-0.97)[d]	0.87 (0.78-0.98)[d]
Q4 (>12.34)	0.81 (0.72-0.90)[d]	0.80 (0.78-0.90)[d]
**First transplant waitlisting**		
Q1 (≤8.60)	1.00 (ref.)	1.00 (ref.)
Q2 (8.61-10.20)	0.98 (0.93-1.04)	0.98 (0.93-1.04)
Q3 (10.21-12.33)	0.94 (0.88-0.99)[d]	0.94 (0.89-1.00)
Q4 (>12.34)	0.90 (0.85-0.95)[d]	0.90 (0.84-0.95)[d]

HR, hazard ratio.
[a]Fully adjusted models (including demographics, clinical characteristics, and facility characteristics; see Table 2). From mixed-effects Poisson models including facility as a random effect.
[b]Using Cox models, without accounting for facility-level shared frailty (models do not converge).
[c]Fine and Gray method used to account for competing risks, which included: mortality=receipt of a kidney transplant, switch to another dialysis modality, or recovery of renal function; transplantation, waitlisting, all-cause hospitalization, and hospital readmission=death, switch to another dialysis modality, or recovery of renal function; and cause-specific hospitalization=death, switch to another dialysis modality, recovery of renal function, or hospitalization due to any other cause.
[d]Statistically significant estimate at P<0.05.

© 2024 Plantinga LC et al. *JAMA Network Open.*

eTable 6. Associations of Patient:PCT Ratios With Different Operationalizations of Outcomes: Sensitivity Analyses

Outcome	Excluding events at ::90 days[b] IRR (95% CI)	Event defined by diagnostic code in any position IRR (95% CI)
**Mortality**		
Q1 (≤8.60)		
Q2 (8.61-10.20)		
Q3 (10.21-12.33)		
Q4 (>12.34)		
**First all-cause hospitalization**		
Q1 (≤8.60)	1.00 (ref.)	
Q2 (8.61-10.20)	0.99 (0.96-1.03)	
Q3 (10.21-12.33)	0.99 (0.96-1.03)	
Q4 (>12.34)	1.03 (1.00-1.07)	
**First all-cause readmission**		
Q1 (≤8.60)	1.00 (ref.)	
Q2 (8.61-10.20)	0.99 (0.93-1.05)	
Q3 (10.21-12.33)	1.00 (0.95-1.06)	
Q4 (>12.34)	1.03 (0.97-1.10)	
**First hospitalization due to fluid overload**		
Q1 (≤8.60)	1.00 (ref.)	1.00 (ref.)
Q2 (8.61-10.20)	1.01 (0.94-1.07)	1.00 (0.94-1.05)
Q3 (10.21-12.33)	1.04 (0.97-1.11)	0.98 (0.93-1.04)
Q4 (>12.34)	1.05 (0.99-1.13)	1.03 (0.97-1.08)
**First hospitalization due to sepsis**		
Q1 (≤8.60)	1.00 (ref.)	1.00 (ref.)
Q2 (8.61-10.20)	1.02 (0.96-1.08)	1.00 (0.94-1.05)
Q3 (10.21-12.33)	1.02 (0.96-1.08)	0.98 (0.93-1.04)
Q4 (>12.34)	1.08 (1.02-1.14)[c]	1.03 (0.97-1.08)
**First hospitalization due to vascular access complications**		
Q1 (≤8.60)	1.00 (ref.)	1.00 (ref.)
Q2 (8.61-10.20)	0.97 (0.85-1.12)	0.98 (0.88-1.09)
Q3 (10.21-12.33)	1.08 (0.94-1.23)	1.05 (0.94-1.17)
Q4 (>12.34)	1.22 (1.07-1.40)[c]	1.15 (1.03-1.28)[c]
**First transplant**		
Q1 (≤8.60)		
Q2 (8.61-10.20)		
Q3 (10.21-12.33)		
Q4 (>12.34)		
**First transplant waitlisting**		
Q1 (≤8.60)	1.00 (ref.)	
Q2 (8.61-10.20)	0.99 (0.92-1.06)	
Q3 (10.21-12.33)	0.94 (0.88-1.01)	
Q4 (>12.34)	0.92 (0.86-0.99)[c]	

IRR, incidence rate ratio.
[a]Fully adjusted models (including demographics, clinical characteristics, and facility characteristics; see Table 2). From mixed-effects Poisson models including facility as a random effect.
[b]Because patients had to survive and remain on hemodialysis for 90 days to be included, the results for mortality and transplant are the same as for the primary analysis.
[c]Statistically significant estimate at *P*<0.05.

© 2024 Plantinga LC et al. *JAMA Network Open.*

eFigure 1. Mandated US Interdisciplinary Hemodialysis Care Team

APP, advanced practice provider; LVN/LPN, licensed vocational/practical nurses; PCT, patient care technician; RN, registered nurse. *APPs and LVNs/LPNs are used at some facilities but are not required.

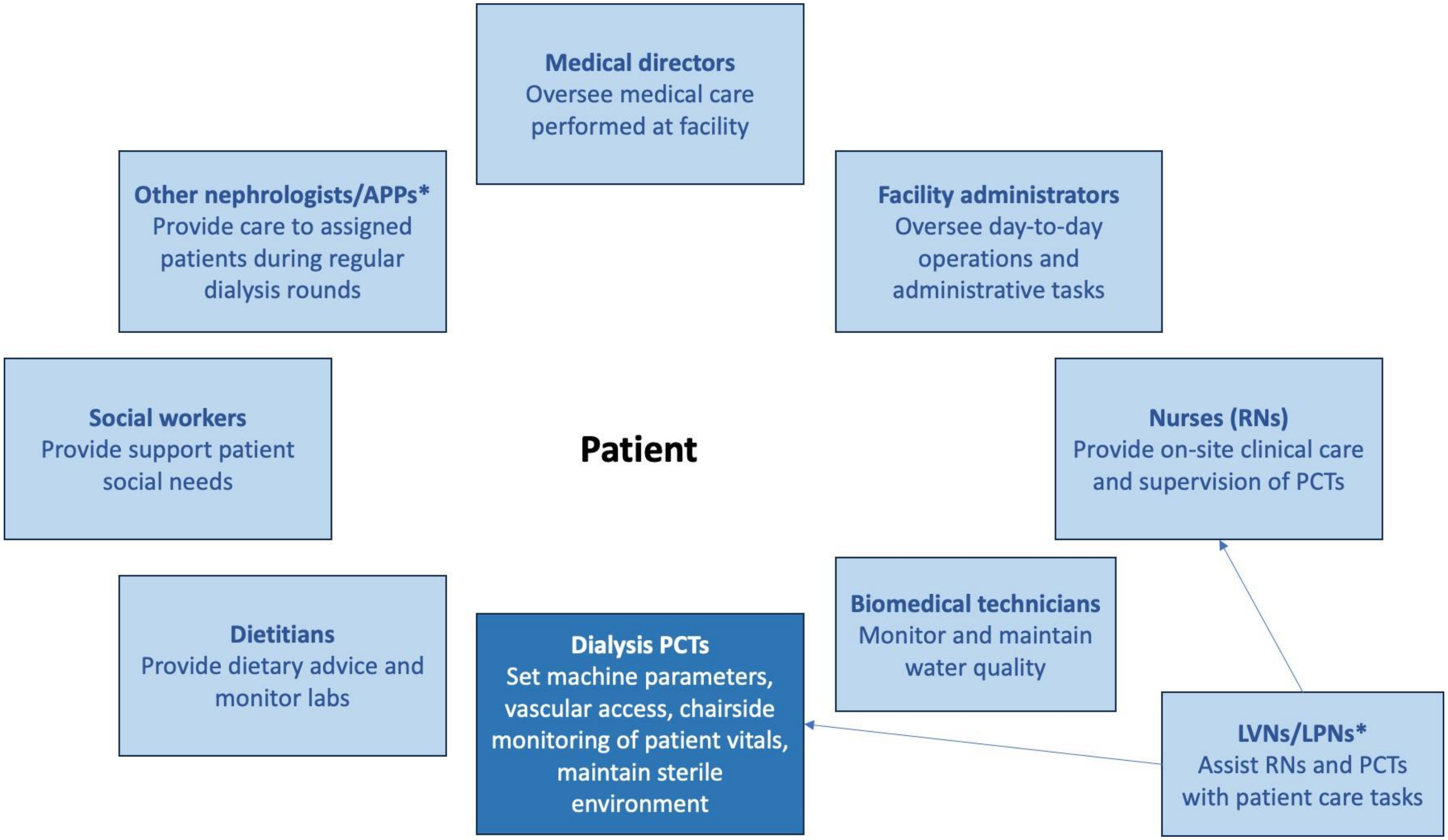

© 2024 Plantinga LC et al. *JAMA Network Open*.

eFigure 2. Selection of Patient Population

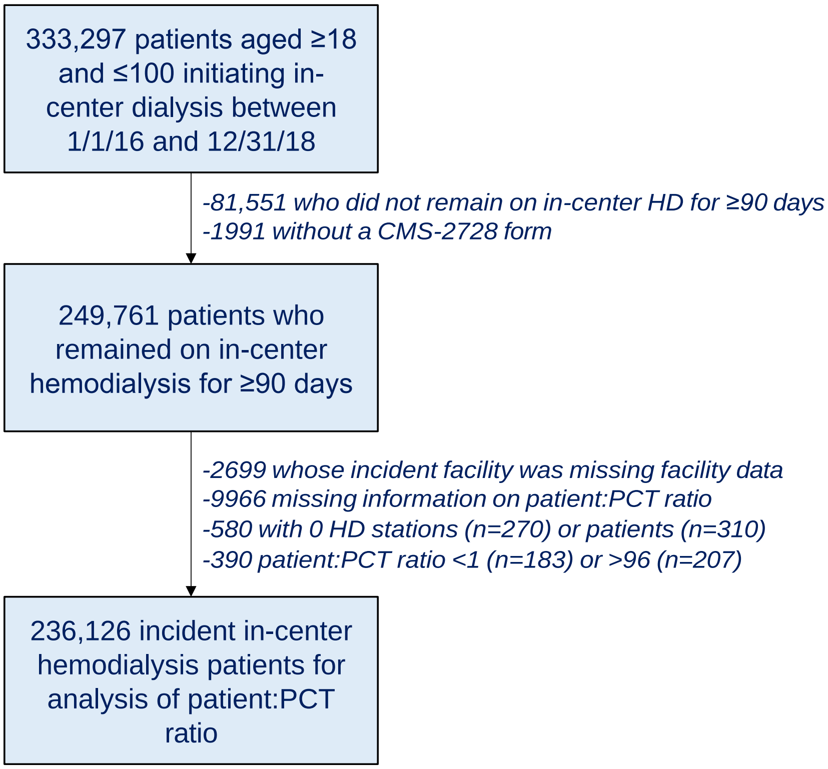

© 2024 Plantinga LC et al. *JAMA Network Open.*

eFigure 3. Patient Follow-Up for Time-to-Event Outcomes

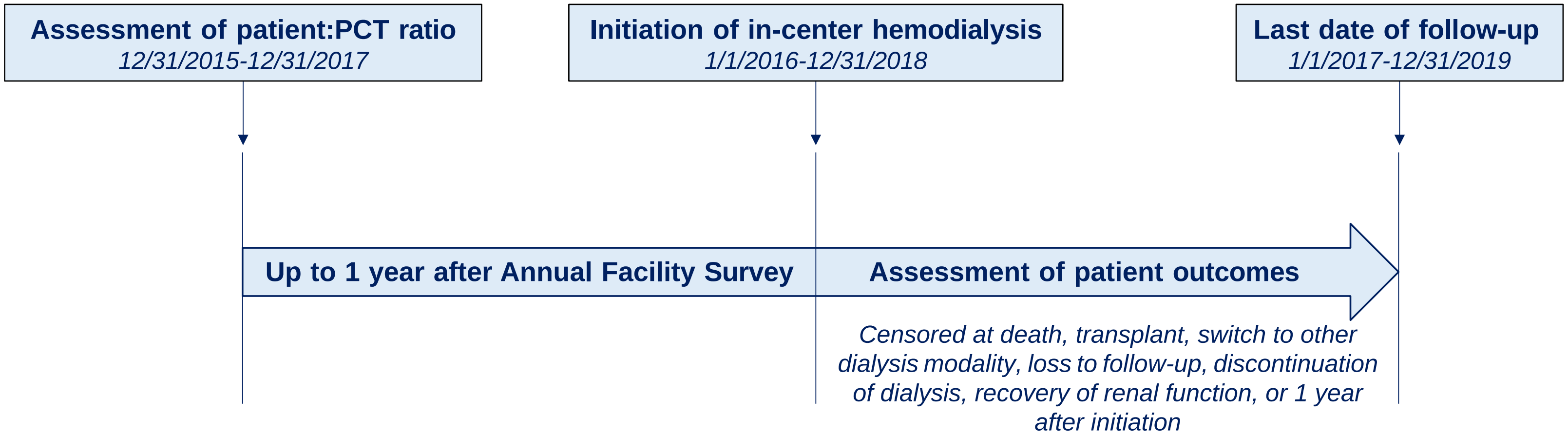

© 2024 Plantinga LC et al. *JAMA Network Open*.

eFigure 4. Distribution of Patient-Level Patient:PCT Ratio
Lines, cutoffs for quartiles.

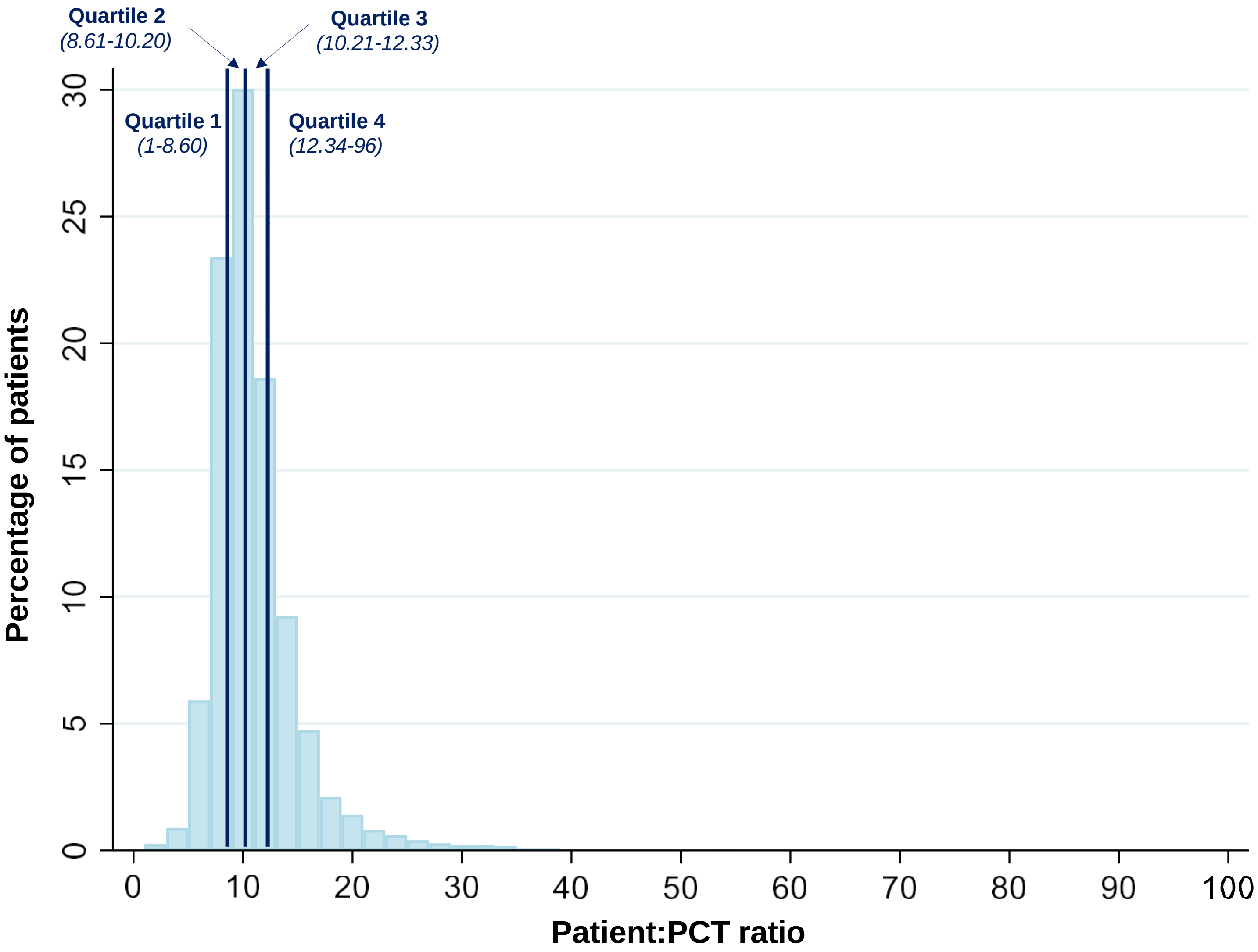

© 2024 Plantinga LC et al. *JAMA Network Open.*
