# Supplementary material for: Patient Care Technician Staffing and Outcomes Among US Patients Receiving In-Center Hemodialysis
Source: JAMA Netw Open. 2024 Mar 8;7(3):e241722. doi: 10.1001/jamanetworkopen.2024.1722 (PMC10924248; doi:10.1001/jamanetworkopen.2024.1722)
Supplement: Supplement 2. — Data Sharing Statement [file jamanetwopen-e241722-s002.pdf]

Data Sharing Statement

Plantinga. Patient Care Technician Staffing and Outcomes Among US Patients Receiving In-Center Hemodialysis. *JAMA Netw Open*. Published March 08, 2024.
doi:10.1001/jamanetworkopen.2024.1722

Data
Data available: Yes
Data types: Deidentified participant data, Data dictionary
How to access data: Researchers can obtain data and dictionaries via request to the United States Renal Data System (USRDS): https://www.niddk.nih.gov/about-niddk/strategic-plans-reports/usrds/for-researchers
When available: With publication

Supporting Documents
Document types: None

Additional Information
Who can access the data: Researchers whose request is approved by the United States Renal Data System.
Types of analyses: Data are released to researchers by the USRDS for the purposes of research and a research proposal is required with submission of a data request.
Mechanisms of data availability: Data are available without cost but require an approved request and fully executed data use agreement (DUA) with USRDS.
Any additional restrictions: Restrictions on reporting of the data are listed on the USRDS DUA. USRDS approval is required for all publications created with their data.
